# The relationship between the systemic immune-inflammation index and hepatitis C virus infection: NHANES 2005 to 2012

**DOI:** 10.1097/MD.0000000000046673

**Published:** 2025-12-19

**Authors:** Yuyu Zeng, Kaige Zhang, Xiaoping Wu

**Affiliations:** a Department of Infectious Disease, The First Affiliated Hospital, Jiangxi Medical College, Nanchang University, Nanchang, Jiangxi Province, China; b Department of Gastroenterology, The Second Affiliated Hospital, Jiangxi Medical College, Nanchang University, Nanchang, Jiangxi Province, China.

**Keywords:** hepatitis C, NHANES, systemic immune-inflammation index

## Abstract

The systemic immune-inflammation index (SII), a contemporary biomarker indicative of inflammatory processes, captures the localized immune reactions and systemic inflammatory responses within the human body. Evidence has established correlations between SII and the incidence as well as the gravity of a range of hepatic pathologies. Nonetheless, the extant literature presents a paucity of studies exploring the SII’s role and its clinical significance in the context of hepatitis C virus (HCV) infection. The objective of this research is to scrutinize the correlation between SII and HCV infection. Our research utilized data extracted from the national health and nutrition examination survey (NHANES). Owing to the asymmetric distribution of SII values, a natural logarithm transformation was implemented to achieve a more normal distribution. A Logit regression analysis was employed to assess the relationship between the natural logarithm of the SII (LN SII) and HCV infection. This cross-sectional analysis included 29,546 participants from 4 cycles of NHANES data. The results of the Logit model indicated that lower values of LN SII were linked to a higher probability of HCV infection. This relationship remained significant in the fully adjusted model (OR = 0.455; 95% CI: 0.381–0.543, *P* <.001). Furthermore, the analysis of the smoothly fitted curve unveiled a relationship resembling an inverted U-shape between LN SII and HCV infection, featuring a turning point value of 6.12. This study established a link between lower LN SII levels and the occurrence of HCV infection. To validate these findings, further large-scale and prospective studies are required.

## 1. Introduction

Hepatitis C, an inflammatory condition of the liver, is precipitated by the presence of the hepatitis C virus (HCV).^[1]^ This viral infection can manifest as either acute or chronic hepatitis, with clinical outcomes varying from mild to severe, persistent conditions such as cirrhosis and hepatocellular carcinoma (HCC). According to a 2022 report by the World Health Organization, an estimated 58 million individuals globally are currently affected by HCV infection, with around 1.5 million new cases diagnosed each year.^[2]^ In 2019, it was estimated that approximately 290,000 individuals succumbed to chronic complications related to HCV, with the majority of these deaths being linked to cirrhosis or HCC.^[2]^ Statistical data indicates that approximately 30% of individuals infected with HCV (ranging from 15 to 45%) will spontaneously clear the virus within 6 months without the need for treatment. In contrast, the remaining 70% (ranging from 55 to 85%) will progress to chronic HCV infection. Among those with chronic infection, the likelihood of developing cirrhosis within 20 years is estimated to be between 15% and 30%.

Prior scholarly work has established a substantial correlation between the “neutrophil-to-lymphocyte ratio” and the “platelet-to-lymphocyte ratio,” linking these ratios to both mortality and the incidence of hepatic decompensation in individuals with hepatitis C.^[3]^ The systemic immune-inflammation index (SII), which amalgamates the neutrophil-to-lymphocyte ratio and platelet-to-lymphocyte ratio indices, is calculated using the formula “platelet count multiplied by neutrophil count divided by lymphocyte count".^[4]^ The SII is considered a consistent and dependable marker of both immune response and systemic inflammation.^[5]^ A variety of studies have emphasized the potential utility of the SII in evaluating disease risk and prognosis, especially in the field of oncology.

For example, the research conducted by Fan and colleagues indicated that the SII serves as a potent predictor of outcomes for individuals who have undergone extensive surgical removal of liver cancer.^[6]^ Jin and team found a significant positive association between elevated SII values and the likelihood of developing prostate cancer.^[7]^ Additionally, it has been demonstrated that SII concentrations are positively correlated with frailty, overall mortality, mortality attributed to cardiovascular diseases, and mortality due to cancer among vulnerable older adults in the United States.^[8]^

Although the link between immune function and HCV infection is extensively documented, a comprehensive assessment of the nexus between the SII and HCV infection remains unexplored. Consequently, the present investigation leverages data from the National Health and Nutrition Examination Survey (NHANES) to conduct an analysis aimed at elucidating the association between the SII and HCV infection.

## 2. Methods

### 2.1. Study participants

**Figure 1.**
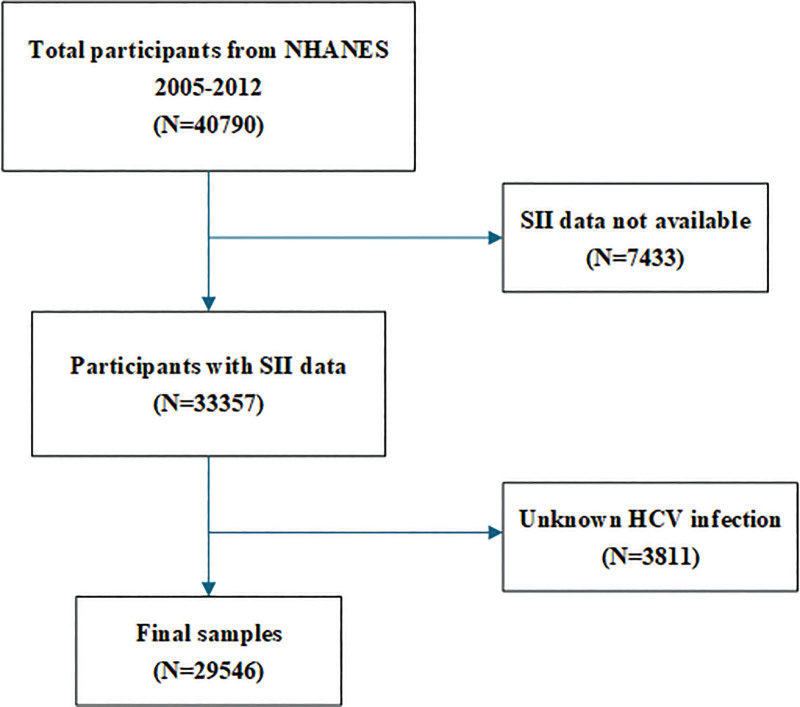
Flowchart of the sample selection from NHANES 2005–2012. NHANES = National Health and Nutrition Examination Survey.

The dataset encompassing all subjects was sourced from the NHANES, a survey that is representative of the entire U.S. demographic and employs sophisticated, stratified probabilistic sampling methods to collect data pertaining to dietary habits and health conditions. Managed by the Centers for Disease Control and Prevention, NHANES undergoes periodic updates every 2 years. In the context of this research, information from NHANES cycles between the years 2005 and 2012 (encompassing 2005–2006, 2007–2008, 2009–2010, and 2011–2012) was extracted for analysis. Consent was obtained from all individuals involved, with the research having been sanctioned by the ethical review committee of the National Center for Health Statistics. The study was conducted in accordance with the guidelines of the Declaration of Helsinki. Out of 40,790 eligible individuals, 7433 were excluded due to missing SII data, and 23,841 were excluded due to unavailable HCV infection status. The final study sample consisted of 29,546 participants. The process of selecting the sample is illustrated in Figure 1.

### 2.2. Study variables

Within the scope of this investigation, HCV infection was identified based on the presence of either anti-HCV antibodies or HCV RNA, as indicated by positive test results.^[9]^ The independent variable under consideration was the SII, with HCV infection status serving as the dependent variable. Hematological parameters, encompassing lymphocyte, neutrophil, and platelet counts, were ascertained through the utilization of an automated hematology analyzer. The calculation of the SII was executed by employing the formula: the product of platelet and neutrophil counts, divided by the lymphocyte count.^[10]^

Due to the skewness observed in the distribution of SII, a natural logarithmic (LN) transformation was employed to approximate a normal distribution. To adjust for the potential confounding effects of other variables on HCV infection, relevant covariates were incorporated into the analysis. The inclusion of these covariates was guided by findings from previous studies on the topic.^[11–13]^ In conclusion, factors such as sex, age, ethnicity, income-to-poverty ratio, body mass index (BMI), smoking habits, alcohol intake, diabetes, and hypertension were identified as potential confounding variables to be included in the study’s analysis.

### 2.3. Statistical analysis

In this research, the computation of statistical analyses and the depiction of graphical data were executed utilizing R software, version 4.0.3. Initially, the demographic and clinical attributes of the study cohort were delineated based on their HCV infection status. Numerical variables were encapsulated as weighted averages complemented by standard errors, and discrete variables were articulated as weighted fractions. To scrutinize the correlation between the LN SII and HCV infection, a multivariate weighted logistic regression model was engaged. The exploration of a potential non-linear relationship was augmented through the application of smooth curve fitting and threshold effect analysis. Additional analyses were conducted to assess subgroups, stratified by factors such as age, gender, ethnicity, and BMI. The threshold for statistical significance was set at *P* <.05.

## 3. Results

### 3.1. Baseline characteristics of participants

This study included a total of 29,546 participants, with the flowchart depicting the enrolled population presented in Figure 1. In the cohort, 48.8% were males, having an average age of 40.4 ± 20.3 years. The average SII was recorded at 548.8 ± 341.7. Additionally, 1.3% of the subjects were identified with HCV infection, aligning with the prevalence estimates of 0.3 to 2.0% among the broader U.S. population.^[14]^ Table 1 delineates the clinical profiles of the subjects, categorized by their HCV infection status. Notable correlations were identified between HCV infection status and several variables, such as age, gender, ethnicity, income-to-poverty ratio, BMI, alcohol intake, smoking, hypertension, and diabetes (*P* <.05). Subjects with HCV infection were generally older, exhibited a higher BMI, were predominantly male and non-Hispanic white, and had a greater incidence of smoking, alcohol consumption, hypertension, and diabetes compared to those without HCV infection (*P* <.05).

**Table 1:** The weighted characteristics of the study participants based on hepatitis C virus infection status.

Characteristics	HCV infection		*P*-value
	No	Yes	
No. of participants	29,156	390	<.001
Age (year)	37.70 ± 22.83	51.20 ± 10.96	<.001
PIR	2.20 ± 1.67	1.71 ± 1.45	<.001
BMI (kg/m^2^)	26.74 ± 7.31	28.28 ± 6.80	<.001
Waist circumference (cm)	91.22 ± 19.47	98.95 ± 15.79	<.001
SII	537.73 ± 378.53	456.52 ± 325.31	<.001
LN SII	6.13 ± 0.56	5.90 ± 0.69	<.001
Gender (%)			
Male	14,398 (49.38%)	246 (63.08%)	<.001
Female	14,758 (50.62%)	144 (36.92%)	
Race (%)			
Mexican American	9.41	6.06	<.001
Other Hispanic	5.26	4.76	
Non-Hispanic White	67.30	66.58	
Non-Hispanic Black	11.33	18.85	
Other Race	6.69	3.75	
Drink (%)			
Yes	13,134 (45.05%)	303 (77.69%)	<.001
No	5313 (18.22%)	49 (12.56%)	
Unclear	14 (0.05%)	1 (0.26%)	
Missing	10,695 (36.68%)	37 (9.49%)	
Hypertension (%)			
Yes	6967 (23.90%)	169 (43.33%)	<.001
No	15,642 (53.65%)	221 (56.67%)	
Unclear	38 (0.13%)	0 (0.00%)	
Missing	6509 (22.32%)	0 (0.00%)	
Diabetes (%)			
Yes	2379 (8.16%)	53 (13.59%)	<.001
No	26,339 (90.34%)	330 (84.62%)	
Borderline	416 (1.43%)	6 (1.54%)	
Missing	22 (0.08%)	1 (0.26%)	
Smoke (%)			
Yes	9025 (30.95%)	330 (84.62%)	<.001
No	10,912 (37.43%)	57 (14.62%)	
Unclear	13 (0.04%)	0 (0.00%)	
Missing	9206 (31.57%)	3 (0.77%)	

Mean ± SD for continuous variables: The *P*-value was calculated by the weighted linear regression model. % for categorical variables: The *P*-value was calculated by a weighted χ^2^ test.

BMI = body mass index, HCV = hepatitis C virus, LN = logarithmic, PIR = income to poverty ratio, SII = systemic immune-inflammation index.

### 3.2. The correlation between the LN SII and the condition of HCV infection

Our examination discloses an inverse relationship between the LN SII and the likelihood of HCV infection, where lower LN SII levels correspond to an increased likelihood of infection. This association remained statistically significant in all examined models: The initial model (OR = 0.502; 95% CI: 0.427–0.592; *P* <.001), the semi-adjusted model (OR = 0.525; 95% CI: 0.448–0.617; *P* <.001), and the comprehensively adjusted model (OR = 0.455; 95% CI: 0.381–0.543; *P* <.001) were all evaluated. Sensitivity analyses employed LN SII quartiles. In the comprehensively adjusted model, the ORs for quartiles Q1, Q2, Q3, and Q4 were 1.00, 0.38 (0.28, 0.52), 0.42 (0.31, 0.56), and 0.34 (0.25, 0.47), respectively. Contrasting Q1 with Q4, individuals in Q4 showed a 66% decrease in the likelihood of HCV infection (*P* for trend <.001). Moreover, the trend *P*-values across the 3 models were below .05, signifying statistical significance (Table 2).

**Table 2:** The association between the natural logarithm of systemic immune-inflammation index and hepatitis C virus infection.

Exposure	Crude model OR (95% CI) *P*-value	Model 1 OR (95% CI) *P*-value	Model 2 OR (95% CI) *P*-value
LN SII	0.50 (0.43, 0.59)*	0.53 (0.45, 0.62)*	0.46 (0.39, 0.55)*
LN SII quartiles			
Q1	Reference	Reference	Reference
Q2	0.42 (0.32, 0.56)*	0.43 (0.32, 0.57)*	0.38 (0.28, 0.52)*
Q3	0.47 (0.36, 0.62)*	0.47 (0.36, 0.62)*	0.42 (0.31, 0.56)*
Q4	0.45 (0.34, 0.59)*	0.42 (0.32, 0.56)*	0.34 (0.25, 0.47)*
*P* for trend	<.001	<.001	<.001

Model 1 was adjusted for age, gender, and race; Model 2 was adjusted for age, gender, race, PIR, BMI, smoking status, alcohol consumption, diabetes, and hypertension.

BMI = body mass index, CI = confidence interval, LN = logarithmic, PIR = income-to-poverty ratio, OR = odds ratio, SII = systemic immune-inflammation index.

* *P* < .001; *P* < .05 was considered statistically significant.

### 3.3. Stratified analysis

**Figure 2.**
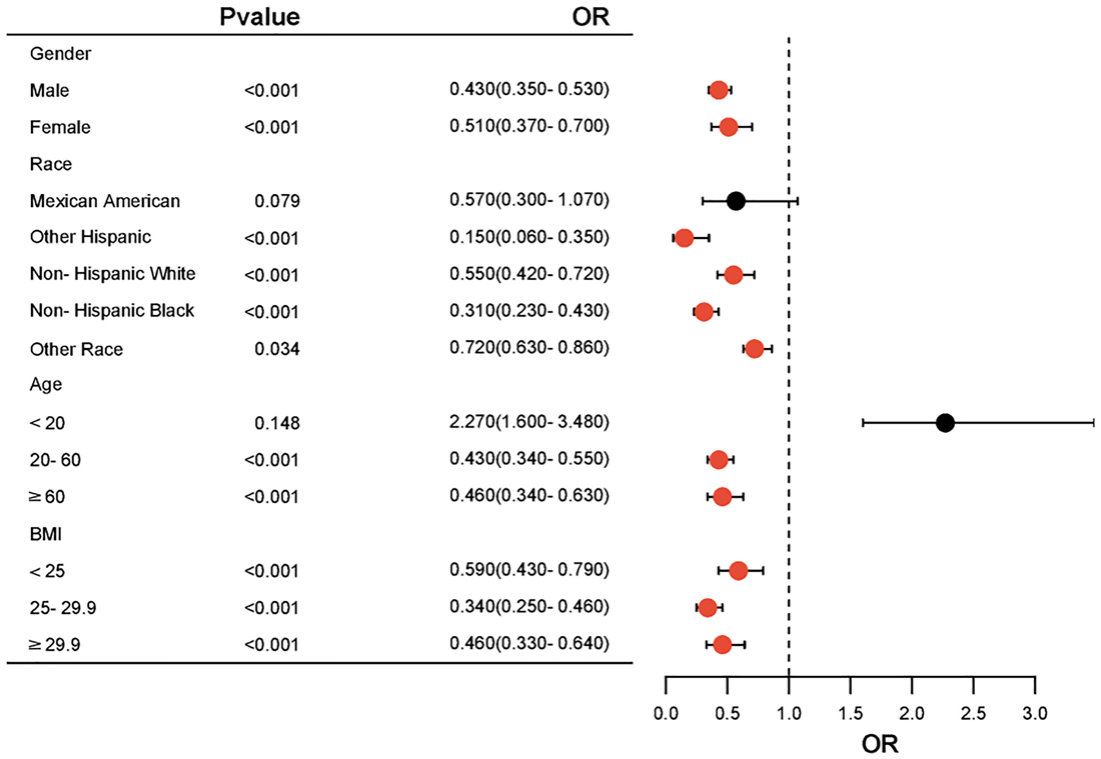
Forest plot of subgroup analysis on the association between LN SII and HCV infection. HCV = hepatitis C virus, LN = logarithmic, SII = systemic immune-inflammation index.

In order to delve deeper into the possible influence of confounding variables on the link between LN SII levels and HCV infection, the study population was stratified based on gender, ethnicity, age, and BMI. The subsequent analysis within these subgroups indicated an inverse relationship between LN SII levels and HCV infection across the majority of categories, except for those aged <20 years and Mexican American (*P* <.05) (Fig. 2).

### 3.4. Smooth fitting curves and threshold effect analyses

**Figure 3.**
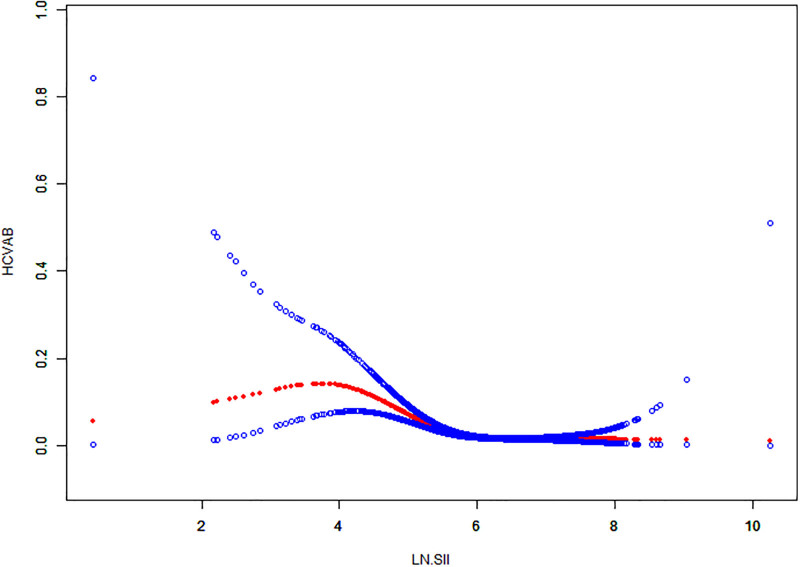
The association between LN SII and HCV infection. HCV = hepatitis C virus, LN = logarithmic, SII = systemic immune-inflammation index.

**Figure 4.**
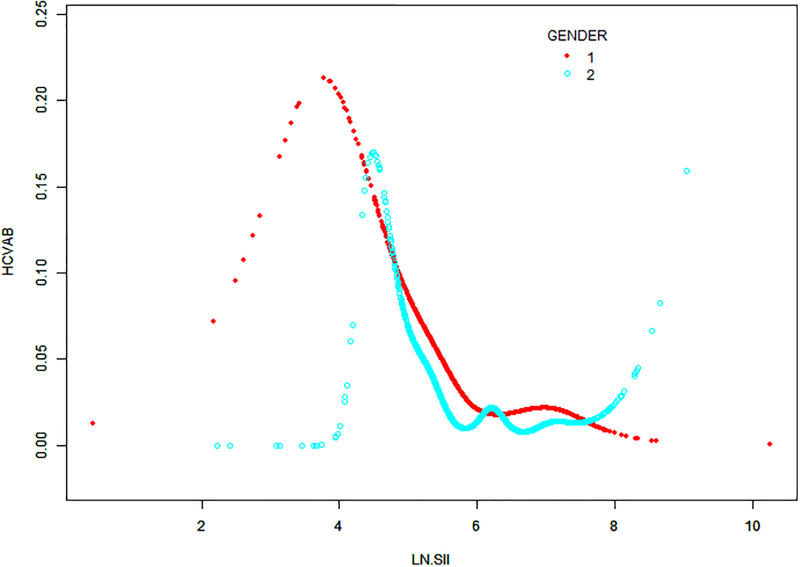
The association between LN SII and HCV infection stratified by gender. Note: 1, Male; 2, Female. HCV = hepatitis C virus, LN = logarithmic, SII = systemic immune-inflammation index.

A non-linear relationship analysis using smooth curve fitting was performed to scrutinize the association between the LN SII and HCV infection (Fig. 3), uncovering an inverted U-shaped pattern with a point of inflection at 3.92. When stratified by gender, similar inverted U-shaped patterns were observed in both males and females, with inflection points at 3.65 and 4.45, respectively (Fig. 4). Furthermore, threshold effect analysis indicated that when LN SII exceeded 3.92, it functioned as a protective factor against HCV infection, yielding a HR of 0.42 (0.34, 0.51); *P* <.0001. The uniform pattern of the inverse U-shaped relationship between the LN SII and HCV infection was noted among various gender groups, featuring inflection points at 3.65 for males and 4.45 for females. Once the LN SII surpassed the respective thresholds, the probability of HCV infection decreased progressively, with statistical significance (*P* <.001) (Table 3).

**Table 3:** Analysis of threshold effects of the natural logarithm of systemic immune-inflammation index on hepatitis C virus Infection using linear regression models.

	Adjusted OR (95% CI), *P*-value
LN SII	
Inflection point	
LN SII < 3.92	1.49 (0.26–8.46); .6550
LN SII ≥3.92	0.42 (0.34–0.51)*
Log likelihood ratio	.067
Male	
Inflection point	
LN SII < 3.65	3.26 (0.23–46.00); .3824
LN SII ≥ 3.65	0.38 (0.29–0.48)*
Log likelihood ratio	.024
Female	
Inflection point	
LN SII <4.45	Inf (0.00–Inf) .9834
LN SII ≥4.45	0.46 (0.33–0.66)*
Log likelihood ratio	.066

Age, gender, race, income-to-poverty ratio, body mass index, drinking status, smoking status, hypertension, and diabetes were adjusted.

CI = confidence interval, LN = logarithmic, OR = odds ratio, SII = systemic immune-inflammation index.

* *P* <.0001, *P* <.05 was considered statistically significant.

## 4. Discussion

Immunity and inflammation have long been recognized as being associated with the occurrence and progression of various diseases, particularly infectious diseases. In this cross-sectional analysis utilizing the NHANES database, we identified an independent association between lower ln SII levels and HCV infection in individuals aged 20 and above. This finding was further validated through sensitivity analyses and subgroup analyses.

It should be highlighted that a plethora of prior research has uniformly documented a positive link between the SII and the initiation, progression, and negative sequelae of a spectrum of illnesses. For example, Zhang et al^[15]^ described a significant positive correlation between SII and the controlled-attenuation parameter, with a regression coefficient (β) of 0.006, a 95% confidence interval ranging from 0.001 to 0.010, and a *P*-value of .011

Jin et al^[16]^ identified a positive correlation between SII and the occurrence of renal calculi, with an OR of 1.282, a 95% confidence interval from 1.023 to 1.608, and a *P*-value of .034.Furthermore, analogous results have been noted in conditions including hypertension, diabetes mellitus, abdominal aortic calcification, and congestive heart failure.^[17–20]^ Based on the formula used to calculate SII, it shows a positive correlation with platelets and neutrophils, while being inversely related to lymphocytes. Previous research has indicated that HCV infection is associated with neutrophil dysfunction. Individuals with chronic HCV infection frequently show impaired neutrophil function, characterized by reduced phagocytic activity, a higher count of inactive neutrophils, and diminished activation in response to bacterial stimuli.^[21]^ Furthermore, a community-based study involving 11,239 Taiwanese residents evaluated the relationship between platelet count and HCV viremia, finding that platelets are directly affected by HCV and that HCV RNA is significantly correlated with platelet count.^[22]^ These findings align with the present study, indicating that decreased neutrophil and platelet counts are closely related to HCV infection. However, the exact mechanisms underlying these associations remain unclear, and further in-depth research is required in the future.

This study revealed that individuals infected with HCV were older than their uninfected counterparts (51.20 vs 37.70, *P* <.001). In a retrospective observational study, it was determined that individuals with HCV infection who were 65 years of age or older exhibited a 1.14, 2.44, and 2.09 times greater likelihood of developing cirrhosis, HCC, and experiencing all-cause mortality, respectively, when contrasted with those younger than 65 years (*P* <.05).^[23]^ This may be due to age-related factors that increase the risk of liver disease outcomes in individuals with HCV infection. These factors include reduced resistance to environmental stressors, elevated oxidative stress, decreased hepatic blood flow and mitochondrial function, impaired immune response, and a decline in DNA repair capacity with aging, all of which contribute to a heightened risk of carcinogenesis.^[24,25]^

Another finding of this study is that the proportion of males among HCV infected individuals is higher than that among uninfected individuals (63.08% vs 49.38%, *P* <.001), which is primarily associated with the modes of HCV transmission. In many countries, HCV infection is primarily observed in individuals who inject drugs and those who engage in men who have sex with men behaviors. Statistics show that in the United States, males represent roughly two-thirds to three-quarters of injection drug users,^[26]^ resulting in a significantly higher HCV prevalence rate among males compared to females. This study also found that the BMI of HCV-infected individuals is higher than that of uninfected individuals.

The hepatic plays a pivotal role in metabolic processes, with HCV infection being implicated in the initiation of insulin resistance and hepatic steatosis.^[27]^ Similarly, obesity is recognized as a contributing factor to the advancement of chronic hepatitis C and is correlated with insulin resistance.^[28]^ Moreover, the concurrence of chronic hepatitis C and nonalcoholic fatty liver disease frequently results in accelerated disease progression, heightened susceptibility to HCC, and diminished responsiveness to antiviral therapies.^[29]^ However, the exact mechanisms connecting fatty liver, insulin resistance, and the progression of HCV disease remain unclear.

To summarize, this research represents a pioneering effort in investigating the correlation between the SII and HCV infection. The findings indicate a negative correlation between the LN SII and the probability of HCV infection, such that a decrement of one unit in LN SII is associated with a 54% escalation in the risk of infection. Moreover, the correlation between LN SII and HCV infection is nonlinear, with threshold effect analysis disclosing an inverted U-shaped curve, peaking at an inflection point of 3.92. Beyond this threshold, the susceptibility to HCV infection markedly diminishes. These insights propose that diminished LN SII values could be indicative of an elevated risk for HCV infection. Given that LN SII is ascertainable through standard blood assessments, it is advisable for individuals exhibiting reduced LN SII values to engage in periodic HCVAB screening, subsequent to which HCV RNA testing and pertinent antiviral interventions may be warranted, thereby aiding in the realization of the World Health Organization’s objectives to eradicate viral hepatitis by the year 2030.^[2,30]^

This research possesses distinct merits. Initially, the correlation between the LN SII and HCV infection was investigated utilizing an extensive, demographically representative data set. Subsequently, a multitude of pertinent confounding variables were accounted for, and sensitivity analyses were performed to bolster the reliability of our findings. Nevertheless, the study is not without its constraints. Principally, as a cross-sectional investigation, it is limited to indicating an association rather than establishing causation between LN SII and HCV infection. Additionally, the generalizability of the findings to populations outside the US may be limited due to the data’s geographical specificity. Furthermore, despite adjustments for numerous potential confounding elements, the lingering possibility of unmeasured confounding cannot be entirely discounted. Lastly, the lack of data on HCV treatment status and detailed liver disease staging in our dataset prevented us from conducting stratified analyses based on these factors. Future prospective studies that incorporate such detailed clinical information are warranted to validate our findings and to further explore the relationship between the SII and HCV-related disease progression and treatment outcomes.

## Acknowledgments

The study was supported by the Natural Science Foundation of Jiangxi Province, China [20212ACB206010] and Health Commission of Jiangxi Province, China (20202097). Concept, design and drafting of the manuscript (Yuyu Zeng); statistical analysis and data acquisition (Kaige Zhang); critical revision of the manuscript for important intellectual content and obtained funding (Xiaoping Wu). All authors read and approved the final manuscript.

## Author contributions

**Conceptualization:** Yuyu Zeng.

**Data curation:** Kaige Zhang.

**Funding acquisition:** Xiao Ping Wu.

**Writing – original draft:** Yuyu Zeng.

**Writing – review & editing:** Xiao Ping Wu.
